# Age-Related Low Bone Mineral Density in C57BL/6 Mice Is Reflective of Aberrant Bone Morphogenetic Protein-2 Signaling Observed in Human Patients Diagnosed with Osteoporosis

**DOI:** 10.3390/ijms231911205

**Published:** 2022-09-23

**Authors:** Daniel Halloran, Venu Pandit, Connor MacMurray, Victoria Stone, Kailey DeGeorge, Mark Eskander, Denise Root, Sean McTague, Heather Pelkey, Anja Nohe

**Affiliations:** 1 Department of Biological Sciences, University of Delaware, Newark, DE 19716, USA; 2 Delaware Orthopaedic Specialists, Newark, DE 19713, USA; 3 Orthopedic Surgery, ChristianaCare Hospital, Wilmington, DE 19801, USA; 4 Orthopedic Surgery, ChristianaCare Hospital, Newark, DE 19716, USA

**Keywords:** osteoporosis, osteoblasts, osteoclasts, C2C12 cells, CK2, CK2.3, BMP-2, C57BL/6 mice

## Abstract

Osteoporosis (OP) is a bone disorder characterized by decreased bone mineral density (BMD). Bone Morphogenetic Protein-2 (BMP-2) injections are used to promote bone formation in OP patients. However, patients are unresponsive to BMP-2 while displaying an upregulation of BMP Receptor Type 1a (BMPRIa) and protein kinase CK2α (CK2α). A synthetically produced peptide named casein kinase 2.3 (CK2.3) utilizes the BMP-signaling pathway as it enhances osteogenesis of primary osteoblasts isolated from OP patients, whereas BMP-2 does not. Although shown in OP patients, there is currently no reliable mouse model to study BMP-2 and CK2.3 signaling. In this publication, we show that BMPRIa was required for CK2.3-mediated osteogenesis in C2C12 cells with a CRISPR-Cas9-mediated gene knockout for *BMPRIa*. We utilized the C57BL/6 (B6) mouse strain as an aging-model to study aberrant BMP-2 signaling, demonstrating that, like OP patients, in 15 and 20-month mice, BMP-2 did not increase bone growth and displayed upregulated BMPRIa and CK2α protein expression. Furthermore, CK2.3 enhanced osteogenesis and decreased osteoclastogenesis in all age groups, whereas BMP-2 only increased mineralization in 6-month mice while increasing osteoclast formation in all age groups. These data demonstrated that aging B6 mice were a reliable model and mimicked data obtained from OP patients.

## 1. Introduction

Osteoporosis (OP) is a debilitating bone disorder characterized by a decrease in bone mineral density (BMD) [1,2,3]. Approximately 10 million Americans suffer from OP, with another 18 million at risk [4]. The medical expenses associated with OP are about 19 billion dollars annually, a figure that increases yearly [4,5]. Along with suffering from financial burdens, patients exhibit a lower quality of life, making OP devastating both physically and mentally. In addition, 80% of OP patients are women, typically diagnosed post-menopause [6].

The current treatment options for OP are limited. These therapeutics typically target restoration of the balance between bone forming osteoblasts and bone resorbing osteoclasts [1,7,8,9,10,11]. These drugs are either anabolic, which promote bone growth, or antiresorptive, which limit bone resorption [1,7,10,12,13]. However, many of these drugs lead to fractures, cancer, and necrosis [3,14,15,16,17]. A novel therapeutic, Romosozumab, leads to increased bone growth, but its role in preventing bone resorption is still being investigated [10,18,19,20,21,22]. Likewise, its long-term effects are unknown, and there have been reported cases of osteonecrosis and hepatitis [19,20,21,22]. Thus, a treatment that safely increases osteoblastogenesis and decreases osteoclastogenesis simultaneously is needed.

A promising pathway that can be implicated in the development of a new therapeutic to treat OP is the bone morphogenetic protein (BMP) signaling pathway. The BMP-signaling pathway is typically activated by members of the transforming growth factor-beta (TGB-β) superfamily, including BMP-2 and BMP-4 [23,24,25,26,27,28]. Specifically, BMP-2 has a prominent role in osteogenesis, chondrogenesis, and adipogenesis, and it is also an essential regulator of bone homeostasis [27,28,29,30,31,32,33,34,35,36,37,38]. BMP-2 binds to type I and II serine/threonine kinase receptors, including BMP receptor type Ia (BMPRIa) and type II (BMPRII) [32,37,38,39,40]. After binding, protein kinase CK2 (CK2) is released from BMPRIa, and BMPRII phosphorylates BMPRIa at a glycine/serine rich homeobox domain [9,12,26,41,42,43,44,45]. Additionally, BMPRIa and CK2 colocalize at the plasma membrane in C2C12 cells [46]. In canonical signaling, BMPRIa phosphorylates the downstream proteins Smad1/5/8, which then recruit Smad4 and translocate to the nucleus to function as transcription factors [28,47,48,49]. In addition, the BMPRs can also activate the mitogen-activated protein kinase (MAPK) and phosphatidylinositol 3-kinase (PI3K) signaling pathways in non-canonical signaling to enhance cell proliferation and survival [9,41].

Due to BMP-2′s vast functionality, the Food and Drug Administration (FDA) approved its use in 2002 during anterior lumbar interbody fusion (ALIF) and maxillofacial reconstructive surgeries [50,51,52]. However, although restoring bone density, BMP-2 injections led to many side-effects including ectopic bone formation, cervical spinal swelling, enhanced osteoclastogenesis, and retrograde ejaculation [53,54,55,56,57,58,59,60]. In addition, osteoblasts isolated from osteoporotic patients did not respond to BMP-2 stimulation, even though there was an upregulation of BMPRIa and CK2α compared to control patients [42,61]. Therefore, usage of BMP-2 in clinical settings is severely limited. However, the BMP-signaling pathway may still be utilized to create novel drugs that display fewer adverse side-effects.

By using the CK2 phosphorylation site on the BMPRIa receptor, a novel synthetic peptide named casein kinase 2.3 (CK2.3) binds to CK2 and prevents its association with BMPRIa [9,26,41,42,46,62,63]. CK2.3 is a 29 amino acid long peptide that contains the SLKD phosphorylation site on BMPRIa and the Antennapedia homeodomain sequence for cellular uptake [9,12,26,41,42,44,46,48,61,62,64,65]. This leads to the activation of the BMP-signaling pathway, primarily the non-canonical pathway, without endogenous BMP-2 [9,46]. Furthermore, CK2.3 is reliant on the presence of BMPRIa as transfection of BMPRIa siRNA into C2C12 cells completely inhibits CK2.3-mediated osteogenesis [43]. CK2.3 enhances osteogenesis both in vivo and in vitro and is uptaken by cells, predominantly by caveolae [62]. Similarly, CK2.3 increases bone formation and osteoblastic markers in cells isolated from OP patients, whereas this response is not seen upon BMP-2 stimulation [42,61]. However, due to the medical history, treatments, and genetics, the observed results may be inconsistent between patients. Therefore, a model that eliminates the aforementioned complications and determines the specificity of CK2.3 signaling must be explored. To study the roles of CK2.3 and BMP-2 on osteoblastogenesis and osteoclastogenesis in vivo, we utilize C2C12 cells with BMPRIa knockout (KO) and C57BL/6 (B6) mice as a possible model to study aberrant BMP-2 signaling reflective of OP patients.

Here, we show that C2C12 cells with the BMPRIa KO did not mineralize upon BMP-2 and CK2.3 stimulation, while control cells were still able to mineralize. These data indicate that CK2.3 was specific to BMPRIa, and it did not elicit an osteogenic response without the receptor. This is important for understanding the role of this peptide in vivo. Next, we isolated bone marrow stromal cells (BMSCs) and osteoclast precursors from the femurs and spleens, respectively, of B6 mice aged 6, 15, and 20 months. First, similar to OP patients, BMSCs isolated from 15-month-old mice increased BMPRIa and CK2α activity compared to 6-month-old mice. In addition, microcomputed tomography (µCT) analyses indicated that while femurs isolated from 6-month-old mice increased bone volume/tissue volume (BV/TV) after BMP-2 and CK2.3 injection, the 15- and 20-month-old mice responded positively to only CK2.3 compared to control bones. Next, after differentiating the BMSCs into osteoblasts, the cells isolated from 6-month-old mice secreted a significantly higher area of mineralization when stimulated with BMP-2 and CK2.3 as compared to the unstimulated (US) group. However, osteoblasts isolated from the femurs of 15- and 20-month-old mice secreted a larger area of mineralization after being treated with CK2.3, but not with BMP-2 stimulation, compared to the control. Next, osteoclast precursors were differentiated into osteoclasts. BMP-2 stimulated cells displayed a higher number of osteoclasts, whereas CK2.3 treated cells displayed osteoclast counts similar to US cells in all age groups. Together, these data indicate that BMP-2 enhanced osteoblastogenesis in younger mice but not older mice and increased osteoclastogenesis in all age groups. However, CK2.3 promotes osteoblast formation and activity while simultaneously inhibiting osteoclast formation. For the first time, in a mouse model to investigate aberrant BMP-2 signaling, we show that BMP-2 did not increase bone mineralization in aged-mice and that CK2.3 inhibited osteoclastogenesis. As CK2.3 increased bone formation and activity while decreased osteoclast activity, these data suggest its role as a possible therapeutic to treat OP and show that the B6 mice were a viable model to study aberrant BMP-2 signaling.

## 2. Results

### 2.1. BMPRIa Is Required for CK2.3-Mediated Osteogenesis in C2C12 Cells

**Figure 1:**
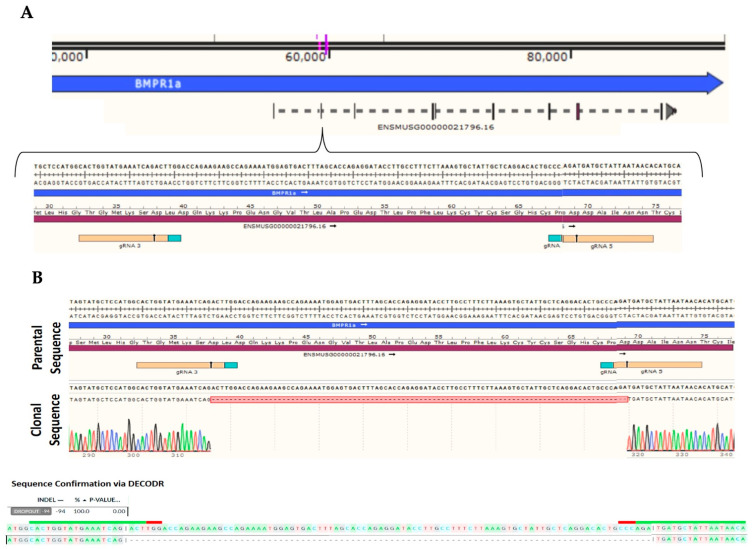
Creation of a BMPRIa Knockout (KO) in the C2C12 cell line. (**A**) In this exon 2 dual guide design, two gRNAs were designed to guide Cas9 to exon 2 within *BMPRIa*. gRNAs 3 and 5 induced a frameshift deletion of 94 base pairs, causing an abrupt stop downstream in the codon. (**B**) In KO clone 4, *BMPRIa* contained a 94 base pair deletion between amino acids 38 (asparagine) and 69 (asparagine). An early stop codon was induced, and DECODR data demonstrated that 100% of the population contained the KO.

**Figure 2:**
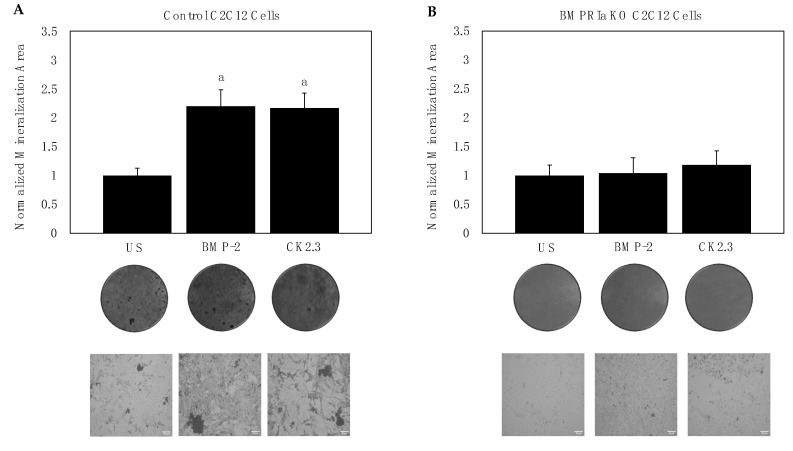
Mineralization deposition of C2C12 cells stimulated with 40 nM BMP-2, 100 nM CK2.3, or left US for five days. (**A**) In positive control C2C12 cells, plates were assayed using the von Kossa method and mineralization deposits were analyzed using ImageJ. BMP-2 and CK2.3 had significantly higher mineralization area compared to the control. (**B**) In C2C12 BMPRIa KO cells, BMP-2 and CK2.3 did not increase mineralization compared to the control. Representative images are displayed underneath the bars. Experiments were repeated in triplicates. Statistical analyses were conducted using the students’ *t*-test and the Tukey–Kramer HSD test. Error bars represent standard error of the mean. Here, letter “a” corresponds to bar 1 (US).

C2C12 cells are murine myoblasts that can be differentiated into functional osteoblasts. These cells express BMPRIa and serve as a cell line model to study BMP-2 and CK2.3-mediated osteogenesis. To determine whether BMP-2 and CK2.3 signal through the BMPRIa receptor, the CRISPR/Cas9-mediated gene knockout for *BMPRIa* in C2C12 cells was utilized to create *BMPRIa* KO clones (Figure 1A,B). First, control C2C12 cells and *BMPRIa* KO clones were plated and stimulated with BMP-2, CK2.3, or no stimulation. The von Kossa assay was then utilized, and we showed that in positive control C2C12 cells, BMP-2 and CK2.3 both induced mineralization (Figure 2A). However, in the *BMPRIa* KO clones, BMP-2 and CK2.3 did not increase mineralization compared to the control group, suggesting BMPRIa was required for BMP-2 and CK2.3-mediated osteogenesis (Figure 2B). After demonstrating that CK2.3 is reliant on BMPRIa, we observed the expression of BMPRIa in B6 mice.

### 2.2. CK2α and BMPRIa Were Overexpressed in 15-Month Mice

**Figure 3:**
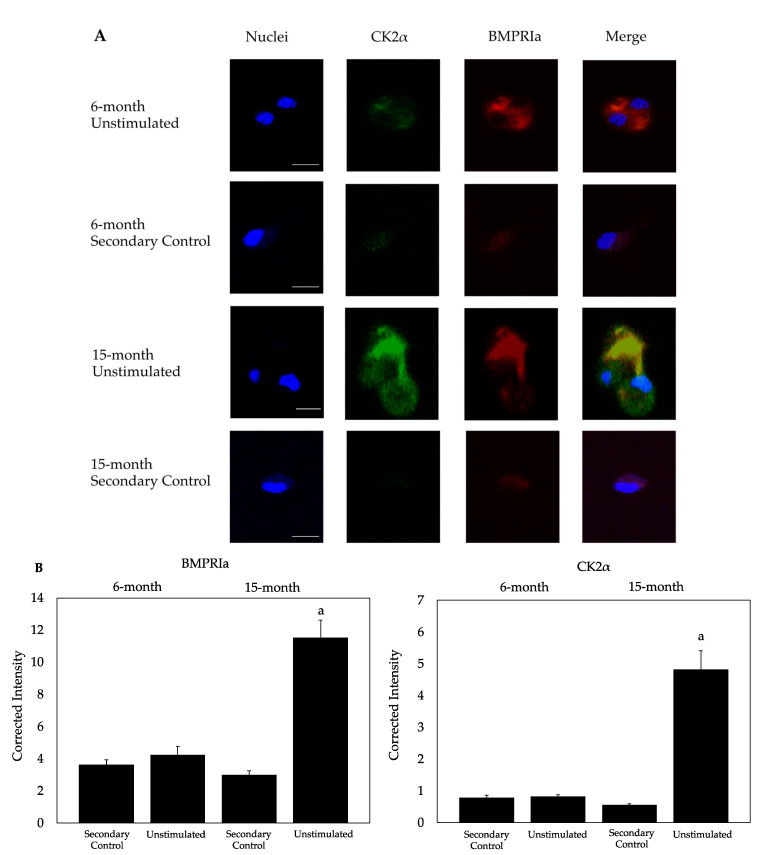
Immunostaining of BMSCs isolated from 6-month-old (*N* = 20) and 15-month-old (*N* = 20) B6 female mice. (**A**) Cells were plated at a concentration of 1x10^6^ cells/mL per well on coverslips in 12-well plates. Confocal microscopic images were obtained at 63x of US and secondary control cells, where the nucleus is stained blue, CK2α is stained green, and BMPRIa is stained red. (**B**) A semi-quantitative analysis was performed in ImageJ to acquire the intensity of fluorescence. BMSCs isolated from US 15-month-old mice displayed an overexpression of CK2α and BMPRIa compared to the other groups. At least 10 images were obtained for each experimental group, and the experiment was conducted twice with three technical replicates. The fluorescence was normalized to the secondary control groups, and data were statistically analyzed using the Tukey–Kramer HSD test, where *p* is set to 0.05 and “a, b, c” lettering denotes statistical significance. Here, letter “a” corresponds to bar 1 (secondary control), letter “b” corresponds to bar 2 (unstimulated), etc. Scale bars are displayed in the nuclei panel and set to 10 μm.

BMPRIa and CK2α are critical for the activation of signaling pathways mediated by BMP-2 and CK2.3. However, in OP patients, the expression BMPRIa and CK2α are upregulated. Here, we utilized B6 mice to observe the expression of these proteins. The BMSCs were isolated, plated at 1 × 10^6^ cells/mL, and immunostained using confocal microscopy (Figure 3A). Compared to the 6-month-old US mice, BMSCs isolated from 15-month-old mice displayed an overexpression of BMPRIa and CK2α, similar to the expression patterns of osteoblasts isolated from OP patients [61]. The pixel intensity was calculated using ImageJ and normalized to the secondary control (Figure 3B). As these proteins are upregulated, we then observed the effect BMP-2 and CK2.3 exerted on the BV/TV of mice.

### 2.3. BMP-2 Did Not Increase BV/TV in 15- and 20-Month-Old Mice upon µCT Analysis

**Figure 4:**
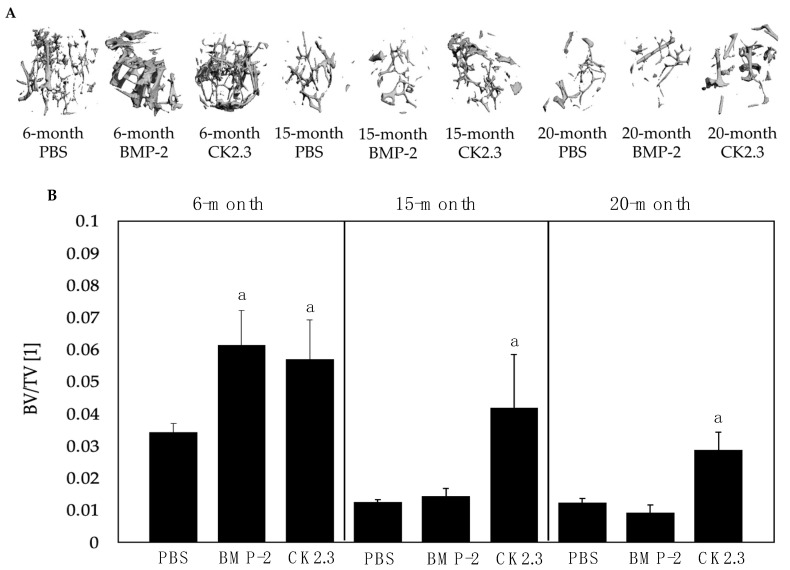
µCT analysis of trabeculae of mice femurs. (**A**) µCT images were obtained of femurs isolated from 6-, 15-, and 20-month-old mice stimulated with PBS, BMP-2, and CK2.3 (*N* = 5/experimental group); 255 scans were taken of each bone and were reconstructed using a 200–1000 threshold. The 3D images were analyzed to obtain BV/TV. Representative images are displayed. (**B**) Bars charts were constructed displaying the mean BV/TV for each experimental group. Error bars represent standard error of the mean, and “a” signifies statistical significance and represents bar 1 (PBS). Scans were conducted in duplicate and statistical analyses were performed using the Fisher’s Exact statistical test.

BV/TV provides an indication of the strength of the bone, which naturally decreases with age. This can be assessed using µCT, which can be utilized after mice are stimulated to obtain bone morphology data. Here, right femurs were isolated from mice injected with PBS, BMP-2, or CK2.3 at their 6 months, 15 months, and 20 months of age and analyzed using the Scanco35 µCT device. Upon analysis, it was revealed that while BMP-2 and CK2.3 both increased the BV/TV of 6-month-old mice compared to the PBS group, only CK2.3 increased the BV/TV of 15- and 20-month-old B6 mice (Figure 4). Statistical analyses were obtained using the Fisher’s Exact statistical test with a *p*-value significance set to 0.05. After determining the effects of BMP-2 and CK2.3 on bone morphology, we then observed the potential of these cells to mineralize bone matrices after stimulation.

### 2.4. BMP-2 and CK2.3 Enhanced Bone Mineralization of 6-Month-Old Mice, While Only CK2.3 Increased Mineralization in 15- and 20-Month-Old Mice

**Figure 5:**
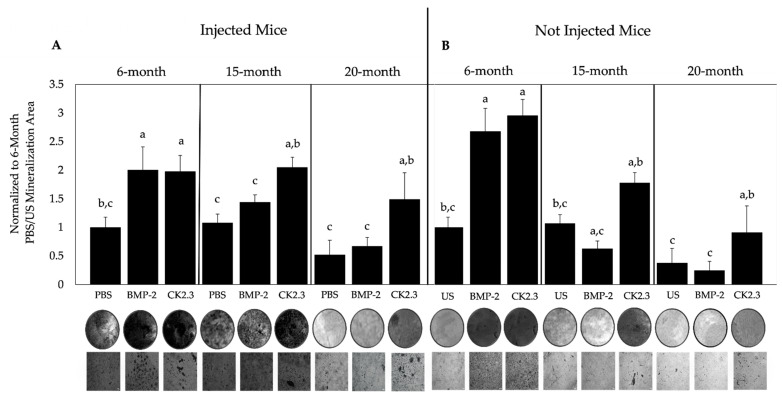
Mineralization deposition of BMSCs isolated from injected and not injected 6-, 15-, and 20-month-old B6 mice. (**A**) Mice were injected with PBS, 5 μg/kg BMP-2, or 2.4 μg/kg CK2.3 for five days. (**B**) Cells were isolated from US mice and stimulated with 40 nM BMP-2, 100 nM CK2.3, or left US. All BMSCs were assayed using the von Kossa method and mineralization deposits were analyzed using ImageJ. Representative images of dishes and magnified deposits are displayed underneath the bars. BMP-2 and CK2.3 had significantly higher mineralization area compared to PBS and US in 6-month-old mice and only CK2.3 significantly increased mineralization compared to PBS and US in 15- and 20-month-old mice. Experiments were repeated in triplicates with five mice per group. Statistical analyses were conducted using the students’ *t*-test and the Tukey–Kramer HSD test. Error bars represent standard error of the mean, and “a, b, c” signifies statistical significance and represents bars 1, 2, and 3 of each group. Scale bars are set to 10 μm.

BMSCs reside in the bone marrow and can differentiate into osteoblasts to form new bone. Therefore, the mineralization areas of these cells isolated from the mice can be assessed. The left femurs of all mice injected with PBS, BMP-2, or CK2.3 were obtained and flushed to isolate BMSCs. The BMSCs were grown in osteoblast differentiation media and assayed using a von Kossa assay. In addition, BMSCs from the femurs of non-injected (negative control) mice were collected. These BMSCs were stimulated with either 40 nM BMP-2, 100 nM CK2.3, followed by the von Kossa assay. The BMSCs isolated from the 6-, 15-, and 20-month-old B6 mice significantly increased bone mineralization after CK2.3 stimulation compared to the control, but only BMSCs from 6-month-old mice responded to BMP-2 in both injected and non-injected mice (Figure 5A,B). Since BMP-2 is not functional in 15- and 20-month-old mice, we then observed its role in osteoclastogenesis.

### 2.5. BMP-2 Increased Osteoclastogenesis, While CK2.3 Decreased Osteoclastogenesis, in All Mice

**Figure 6:**
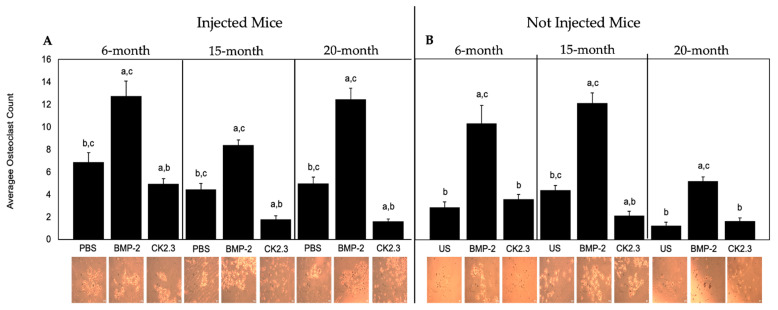
Osteoclastogenesis in mice determined by a TRAP assay. (**A**) The average osteoclast counts of cells isolated from 6-, 15-, and 20-month-old mice injected with BMP-2, CK2.3, or PBS were obtained. (**B**) Osteoclasts isolated from not injected mice stimulated with BMP-2, CK2.3, or left US. Ten random images were taken of each well with a 10X objective and counted independently by three researchers. Representative images are displayed underneath the bars. In all age groups, BMP-2 significantly increased osteoclastogenesis, while CK2.3 prevented osteoclastogenesis, compared to the control groups. Data were analyzed using the students’ *t*-test and Tukey–Kramer HSD statistical tests. All experiments were conducted in technical triplicates. Error bars represent standard error of the mean, and “a, b, c” signifies statistical significance and represents bars 1, 2, and 3 of each group. Scale bars are set to 10 μm.

BMP-2 enhances osteoclastogenesis while CK2.3 effectively inhibits the formation of osteoclasts. However, the function of these proteins in aged mice is not established. Here, B6 mice injected with BMP-2, CK2.3, or PBS were sacrificed, and their spleens were harvested to isolate hematopoietic stem cells (HSCs), pre-osteoclasts, and immune cells. These cells were stained using a TRAP assay kit to assess osteoclastogenesis. Mice that were not injected were subjected to the same experimental procedure; however, these isolated cells were stimulated with 40 nM BMP-2, 100 nM CK2.3, or left US. We showed that in 6-, 15-, and 20-month-old B6 mice, BMP-2 significantly increased osteoclastogenesis, while CK2.3 decreased osteoclastogenesis compared to the PBS and US groups as denoted by osteoclast counts (Figure 6A,B). To compare the effect of these proteins on osteoclastogenesis in humans, we then utilized human femoral heads to isolate osteoclasts.

### 2.6. BMP-2 Increased Osteoclastogenesis, While CK2.3 Decreased Osteoclastogenesis, in Cells Isolated from Human Femoral Heads of OP Patients

**Figure 7:**
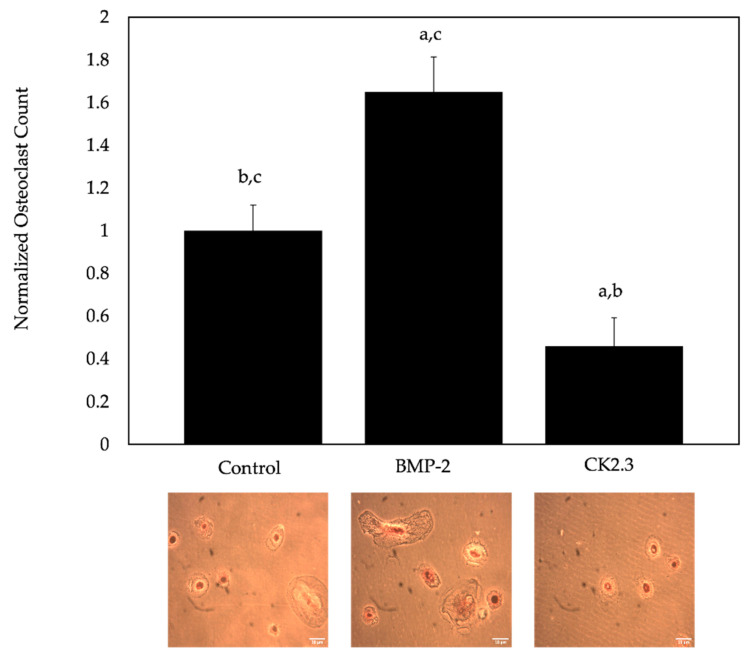
Osteoclastogenesis determined by a TRAP assay. Here, the average osteoclast counts were obtained of cells isolated from human femoral heads of OP female patients. The cells isolated were stimulated with BMP-2, CK2.3, or left US. Ten random images were taken of each well with a 20X objective and counted independently by two researchers. Representative images are displayed underneath the bars. Here, BMP-2 significantly increased osteoclastogenesis, while CK2.3 inhibited osteoclastogenesis, compared to the control group (M-CSF and RANKL only). Data were analyzed using the students’ *t*-test and Tukey–Kramer HSD statistical tests. All experiments were conducted in technical triplicates. Error bars represent standard error of the mean, and “a, b, c” signifies statistical significance and represents bars 1, 2, and 3 of each group. Scale bars are displayed in the bottom right of each image and set to 10 µM.

BMP-2 is known to enhance osteoclastogenesis, but CK2.3 prevents this process. However, the role of these proteins in human patients, especially those diagnosed with OP, is not established. Thus, pre-osteoclasts were isolated from human femoral heads of OP patients and treated with RANKL, BMP-2, or CK2.3. Similar to the mice, CK2.3 inhibited osteoclastogenesis, while BMP-2 increased the formation of osteoclasts compared to the control group (M-CSF + RANKL only, Figure 7).

## 3. Discussion

OP is a devastating bone disorder characterized by a low BMD caused by an imbalance between osteoblasts and osteoclasts. Recombinant human BMP-2 (rhBMP-2) is approved by the FDA due to its versatile roles in bone production and maintenance. However, its osteogenic potential is severely limited in OP patients and leads to several side-effects. A novel peptide, CK2.3, was designed to activate BMP-signaling without exogenous BMP-2. Likewise, CK2.3 has been shown to increase bone formation in both control and OP patients, as well in other models [9,12,26,41,46,61]. While CK2.3 is functional, it is unclear whether its osteogenic potential is reliant on the presence of BMPRIa as transfection of BMPRIa siRNA inhibited its activity [43]. This paper aimed to observe the role of CK2.3 in C2C12 cells with BMPRIa KO to determine if BMPRIa is required for its function.

In addition, we explored the osteogenic activity of BMP-2 and CK2.3, as this has not been well-established in aging mice with low BMD, such as C57BL/6 (B6) mice, which may also display aberrant BMP-2-signaling.

We first utilized CRISPR-Cas9-mediated gene knockout to induce a frameshift mutation in the *BMPRIa* gene of C2C12 cells. C2C12 cells are murine myoblasts capable of differentiating into functional osteoblasts [49]. A dual guide design for exon 2 of *BMPRIa* was developed and created two successful *BMPRIa* KO clones (Figure 1A,B). The *BMPRIa* KO clones were then plated and stimulated with BMP-2 and CK2.3, followed by the von Kossa assay. We demonstrate that BMP-2 and CK2.3 did not induce mineralization in the clones, but this stimulation led to osteogenesis in the positive control C2C12 cells (Figure 2A,B). These data demonstrate that the function of CK2.3 was dependent on BMPRIa and without this receptor present, CK2.3 stimulation did not lead to mineralization, similar to C2C12 cells transfected with BMPRIa siRNA [43].

Next, while previous data demonstrate that osteoblasts isolated from OP patients do not respond to BMP-2, these cells also overexpress BMPRIa and CK2α compared to control cells [61]. Similarly, this overexpression was identified here in the BMSCs isolated from US 15-month-old mice compared to the US 6-month-old mice group (Figure 3A,B). This suggests that aged-mice have a similar responsiveness to BMP-2 as OP patients, indicative aberrant BMP-2 signaling. While the present study did not explore endocytosis and trafficking of BMPRIa bound to BMP-2, future studies should explore this pathway to identify any abnormalities.

While BMP-2 increased the BV/TV of young (6-month-old mice), BMP-2 did not increase bone formation in old B6 mice (15 and 20 months) via µCT analysis (Figure 4), suggesting complications in the BMP-2 signaling pathway. As CK2.3 increased BV/TV in all ages, it is plausible that the BMP-signaling pathway may be successfully activated without exogenous BMP-2 in mice. Further, in the 20-month-old mice stimulated with CK2.3, BV/TV reached levels similar to the 6-month-old PBS control group, suggesting this peptide’s role in rescuing bone formation. The current study did not investigate the rate of mineral deposition, and future studies should utilize dyes, such as calcein, to study this process.

We also showed that BMSCs isolated from 15- and 20-month-old B6 mice did not respond to BMP-2, but CK2.3 injection and stimulation increased mineralization compared to control groups (Figure 5A,B). In addition, both BMP-2 and CK2.3 increased bone mineralization in 6-month-old mice (Figure 5A,B). We utilized the von Kossa assay, which used silver nitrate to bind to components in the bone matrix. These binding sites appeared as dark deposits when exposed to UV, and an increase or decrease in mineral deposition provided an accurate assessment of a change in bone mineralization [66,67].

These results suggest an abnormality within the BMP-2-signaling pathway of aged-mice, which may be partly due to receptor shuffling dynamics or endocytosis. Furthermore, while osteoblasts isolated from OP patients have a decrease in extracellular signal-regulated kinase (ERK) and Smad activity after BMP-2 stimulation, the activity of these proteins has not been elucidated in aged-mice and requires further research [9,12,26,41].

While BMP-2 enhances osteoclastogenesis in humans, this mechanism is not well-understood in mice [59,68]. Furthermore, the role of BMP-2 on the differentiation of osteoclasts in OP patients or aged-mice has not been thoroughly investigated. Previous studies in mice demonstrate that BMP-2 decrease osteoclast formation but increase osteoclast activity (four and eight weeks old) [26,43]. Likewise, while CK2.3 decreases osteoclastogenesis in mice and rats, its role in OP patients or aged mice has not been delineated. Therefore, this study aimed to uncover the mechanism of BMP-2 and CK2.3-mediated osteoclastogenesis. We showed that in both young and old mice, BMP-2 promoted osteoclastogenesis contrarily to previous data, while CK2.3 inhibited the formation of osteoclasts, similar to previous studies (Figure 6A,B) [9,41]. Furthermore, the role of BMP-2 and CK2.3 in osteoclasts isolated from OP patients displayed similar results (Figure 7). The differences in BMP-2 activity may be attributed to the duration of injections or the age of mice. Altogether, our data demonstrated that CK2.3 is capable of decreasing osteoclastogenesis while simultaneously increasing bone formation in old mice and OP patients, whereas BMP-2 actively enhanced osteoclastogenesis in all age groups. These data emphasize the potential role of CK2.3 as a powerful therapeutic in the treatment of OP.

In conclusion, our study demonstrated that CK2.3 is reliant on the presence of BMPRIa to signal. Next, while BMP-2 remained functional in activating BMSCs isolated from young B6 mice, its osteogenic activity was decreased in old mice. We further showed that BMPRIa and CK2α is upregulated in aged-mice, similar to OP patients. Likewise, CK2.3 increased bone mineralization by activating the BMP-signaling pathway in BMSCs isolated from both young and old B6 mice without BMP-2. Contrarily, BMP-2 promoted the differentiation of cells isolated from spleens from young and old mice into functional osteoclasts, suggesting that it retained its osteoclastic activity. Additionally, CK2.3 effectively inhibited osteoclast differentiation in both young and old mice. Altogether, these results suggest that CK2.3 may be used as therapeutic that is both antiresorptive and anabolic. These data demonstrated that CK2.3 can be used to treat OP or be used in future research to develop drugs that can utilize the BMP-signaling pathway.

## 4. Materials and Methods

### 4.1. C2C12 Wildtype and C2C12 BMPRIa Knockout Cell Lines

C2C12 cells, which are immortalized murine myoblast cells, were obtained from American Type Culture Collection (ATCC; Manassas, VA, USA). A subset of the C2C12 cells were targeted using RNP (spCas9 + gRNA) delivery via 4D nucleofection to create a bulk population (ChristianaCare Gene Editing Institute, Newark, DE, USA). Clones 4 and 6 were created using the same set of gRNA’s targeting exon 2 of the *BMPRIa* gene in C2C12. gRNA 3 (CACUGGUAUGAAAUCAGACU) and gRNA 5 (UGUUAUUAAUAGCAUCAUCU) created a 94 base-pair deletion from cut site to cut site, creating a frameshift leading to an early stop codon downstream. The population was sampled at 48 h post-transfection and Sanger sequencing was confirmed to evaluate the amount of frameshift present in the population. One hundred percent of the population displayed a frameshift. The bulk population was single cell sorted into 96-well plates using the Namocell (Namocell, Mountain View, CA, USA). Once the clones became confluent in the 96-well plates, each clone was expanded and sampled. The clones were run through Sanger sequencing and the clones containing a frameshift knockout (KO) were identified, expanded, and cryo-banked. At freezing, a second sample was taken, and confirmatory Sanger sequencing was run. The KO was confirmed via Deconvolution of Complex DNA Repair (DECODR) courtesy of Dr. Byung-Chun Yoo and London McGill of the Gene Editing Institute.

### 4.2. C2C12 Cell Culture

The C2C12 cells were grown with Dulbecco’s Modified Eagle’s Medium (DMEM, Hy-clone, Pittsburgh, PA, USA) supplemented with 10% fetal bovine serum (FBS; Gemini Bioproducts, West Sacramento, CA), 1% penicillin/streptomycin (pen/strep; Fisher Scientific, Pittsburg, PA, USA), and 1% antibiotic/antimycotic (anti/anti; Gemini Bioproducts, West Sacramento, CA, USA) in T25 flasks until 70–75% confluency. Cells were then split into T75 flasks and when confluent, they were plated in 24-well plates at a cell density of 1 × 10^5^ cells/mL.

### 4.3. Mice and Ethical Approval

B6 mice aged 6, 15, and 20 months (*N* = 100) were obtained from Charles River Laboratories (Horsham, PA, USA). After arriving at the mice facility in the Life Science Research Facility (University of Delaware, Newark, DE, USA), the mice were housed four or five per cage and allowed to acclimate for one week. After, 20 mice from each age group were randomly assorted into four categories: unstimulated (US, *N* = 5), phosphate-buffered saline (PBS, *N* = 5) injected, BMP-2 (GenScript, Piscataway, NJ, USA; *N* = 5) injected, and CK2.3 (GenScript, Piscataway, NJ, USA; *N* = 5) injected. The remaining 40 mice were utilized for subsequent experimentation. The mice were weighed prior to any experimentation. Water and food were accessible to the mice throughout the duration of this study. This research study was reviewed and approved by the Institutional Animal Care and Use Committee (University of Delaware, Newark, DE, USA): AUP #1194.

### 4.4. Mice Injections and Organ Isolation

B6 mice were injected with 50 μL of PBS, BMP-2 (5 μg/kg), or CK2.3 (2.3 μg/kg) once per day for five days or were left unstimulated, as previously described [12,26,32,39,41]. BMP-2 and CK2.3 were diluted in PBS as the loading vehicle. After the fifth injection, the mice were housed for two weeks. After two weeks, the mice were weighed and euthanized using CO_2_. After CO_2_ euthanasia, the B6 mice were briefly rinsed in 70% ethanol. Then, the left and right femurs and spleens were harvested. The right femurs were placed in 10% neutral buffered formalin (Sigma Aldrich, St. Louis, MO, USA) for fixation and storage at 4 °C.

### 4.5. Isolation of Bone Marrow Stromal Cells (BMSCs) and Osteoclast Progenitors

To evaluate the osteogenic potential of BMSCs isolated from 6-, 15-, and 20-month-old mice, the cells were isolated from the left femurs of the mice. First, the distal and proximal femoral heads were removed from the femurs to expose the bone marrow. Next, the bones were flushed with alpha minimum essential media (αMEM; Caisson Labs, Smithfield, UT, USA), and the solution was filtered with a 70 μM cell strainer (Stellar Scientific, Baltimore, MD, USA) into 50 mL conical tubes (Cole-Palmer, Vernon Hills, IL, USA). The cells were then centrifuged at 1500 rotations per minute (RPM) for 5 min at 4 °C and resuspended in 5mL of αMEM. Next, to investigate the osteoclastic potential of the isolated osteoclast progenitors, cells were isolated from the spleens of the mice. Spleens were cut in three areas, followed by flushing with αMEM and filtered with 70 μM cell strainers. After, cells were spun down at 1800 RPM at 4 °C and resuspended in 5 mL αMEM supplemented with 25 ng/mL macrophage-colony stimulating factor (M-CSF; Sino Biological, Beijing, China).

### 4.6. Isolation of Pre-Osteoclasts from Human Femoral Heads

To compare the effect of BMP-2 and CK2.3 on osteoclastogenesis in mice, pre-osteoclasts were isolated from human femoral heads. The femoral heads were obtained from the Christiana Care Hospital (Newark, DE, USA or Wilmington, DE, USA) after patients underwent hip replacement surgery. The cells were isolated from OP patients by removing the trabecular bone and flushing the bone marrow to remove adherent cells. After, the cells were filtered with a 70 μM cell strainer and spun down at 1800 RPM for 8.5 min at 4 °C. The cells were resuspended in 5 mL of αMEM supplemented with 25 ng/mL M-CSF. The cells were plated into 12-well plates at a density of 1 × 10^6^ cells/mL.

### 4.7. Immunofluorescent Staining

BMSCs and osteoclast precursors were isolated from the femurs and spleens of B6 mice of 6 and 15 months (*N* = 10/experimental group). The extracted cells were plated at a cell density of 1x10^6^ cells/mL in 12-well plates (Nest Scientific, Woodbridge Township, NJ, USA) on 18 mm diameter rounded coverslips (Catalog #CS-R18-100, Amscope, Irvine, CA, USA) and grown in αMEM supplemented with 10% FBS, 1% pen/strep, and 1% anti/anti solution for one week. The medium was replaced on day three. On day seven, the medium was replaced with osteoblast or osteoclast differentiation media, depending on the cell type. The osteoblast differentiation medium contained αMEM supplemented with 1% pen/strep, 1% anti/anti solution, 200 μL of 25 mg/mL ascorbic acid (Fisher Scientific, Fair Lawn, NJ, USA) and 800 μL of 0.22 g/mL β-glycerol phosphate (Alfa Aesar, Ward Hill, MA, USA). The osteoclast differentiation medium contained αMEM, 25 ng/mL M-CSF, and 50 ng/mL receptor activator of nuclear factor kappa-B ligand (RANKL; Sino Biological, Beijing, China). The pre-osteoclasts were incubated with 25 ng/mL M-CSF for the duration of this study. The medium was replaced every three days for a duration of seven days. On day 14, the medium was aspirated, and the cells were washed with 1X PBS. Cells were then fixed with 4.4% paraformaldehyde (PFA, pH 7.2; Sigma Aldrich, St. Louis, MO, USA) for 15 min at room temperature. After, cells were permeabilized with 0.1% saponin (Sigma-Aldrich, St. Louis, MO, USA) diluted in 1X PBS for 10 min. The cells were then blocked with 3% bovine serum albumin (BSA, Fisher Scientific, Pittsburgh, PA, USA) diluted in 1X PBS, 3% BSA, and 0.1% saponin for 1 h on ice. All cells, except for the secondary control, were incubated with 1:100 dilutions of rabbit polyclonal BMPRIa primary antibody (Catalog #100743-T08; Sino Biological, Beijing, China) and mouse monoclonal CK2α primary antibody (Catalog #sc-373894; Santa Cruz Biotechnology, Dallas, TX, USA) for 1 h on ice. The cells were washed three times with ice cold 1X PBS and incubated with 1:500 dilutions of donkey-anti-mouse (Alexa Fluor^TM^488, Catalog #A21202; Invitrogen, Eugene, OR, USA) and chicken-anti-rabbit (Alexa Fluor^TM^594, Catalog #A21442; Invitrogen, Eugene, OR, USA) in 1X PBS, 3% BSA, and 0.1% saponin for 1 h on ice away from light. After, the wells were washed three times with ice cold PBS, and the nuclei were stained with Hoechst 33,342 (Catalog #AR0039, Bolster Bio, Pleasanton, CA, USA) for 7.5 min. Following nuclear staining, the cells were washed with 1X PBS and mounted on slides with Airvol. The slides were then imaged using the Zeiss LSM880 confocal microscope with Airyscan (Wolf Hall, University of Delaware, Newark, DE, USA) utilizing the 63x objective. The experiment was conducted in triplicate and at least 10 images were acquired for each condition. All data were normalized to the secondary control.

### 4.8. Microcomputed Tomography (µCT)

Right femurs isolated from mice (*N* = 5/experimental group) were fixed with 10% neutral buffered formalin and dehydrated in increasing concentrations of ethanol. After, each femur was analyzed using µCT (Scanco35, Scanco Medical AG, Wangen-Brüttisellen, Switzerland) to obtain BV/TV. The distal ends of the femurs, superior to the growth plate, were observed to obtain trabeculae readings. Here, 255 scans were obtained for each femur, and the femurs were reconstructed. Upon reconstruction, the threshold was set to 200–1000 and three-dimensional images were rendered. All outliers were removed, and the data were averaged to produce a mean BV/TV for each experimental group.

### 4.9. Von Kossa Assay

C2C12 control cells and C2C12 BMPRIa KO cells were grown in 24-well plates at a density of 1 × 10^5^ cells/mL. After reaching confluency, cells were stimulated with 40 nM BMP-2, 100 nM CK2.3, or left US for 5 days. Cells were then fixed with 4.4% PFA for 15 min at room temperature. After, the cells were washed and treated with 5% silver nitrate (Science Company, Lakewood, CO, USA) dissolved in diH2O for one-hour under ultraviolet (UV) light. Then, the plates were washed at least five times with diH2O to remove any excess or unbound silver nitrate. At least 15 images were taken of each well using the Zeiss Axiovert 10 microscope at 10×/12 Achrostigmat objective. The images were converted to 8-bit, a threshold was set to 60, and then they were analyzed using the “analyze particle” function in ImageJ (NIH, Bethesda, MD, USA). The data were normalized to the control.

BMSCs were isolated from femurs of B6 mice (*N* = 5/experimental group). The extracted BMSCs from PBS, BMP-2, and CK2.3-injected mice were plated at a cell density of 1 × 10^5^ cells/mL in 24-well plates or 1 × 10^6^ cells/mL in 12-well plates and grown one-week. The medium was replaced on day three. On day seven, the medium was replaced with the osteoblast differentiation medium, which contained αMEM supplemented with 1% pen/strep, 1% anti/anti solution, 200 μL of 25 mg/mL ascorbic acid and 800 μL of 0.22 g/mL β-glycerol phosphate. The medium was replaced every three days for a duration of seven days. On day 14, the medium was aspirated, and the cells were washed with 1X PBS. The cells then underwent the von Kossa assay as described above.

Similarly, BMSCs isolated from US mice (*N* = 5/group) were seeded in 24-well plates at a density of 1 × 10^5^ cells/mL. The cells were supplemented with αMEM containing 10% FBS, 1% pen/strep, and 1% anti/anti. After three days, BMSCs were stimulated with 40 nM BMP-2, 100 nM CK2.3, or left unstimulated for five days. On the fifth day, a von Kossa assay was performed as described above.

### 4.10. Tartrate Resistant Acid Phosphatase (TRAP) Assay

Here, we utilized the tartrate resistant acid phosphatase (TRAP) assay to assess osteoclast differentiation and activity. Pre-osteoclasts and immune cells, such as macrophages, monocytes, and granulocyte-monocyte progenitors (GMPs), were isolated from the spleens of B6 mice (*N* = 5/experimental group). These cells were plated at a density of 1 × 10^5^ cells/mL in 24-well plates or 1 × 10^6^ cells/mL in 12-well plates supplemented with αMEM containing 10% FBS, 1% pen/strep, and 1% anti/anti solution. After three days, the medium was replaced with osteoclast differentiation media containing αMEM, 25ng/mL M-CSF, and 50 ng/mL RANKL. The osteoclast medium was replaced every three days and on day 10, the medium was removed, and the cells were washed with 1X PBS. Next, cells were fixed with 4.4% PFA for 15 min at room temperature. Then, after washing with 1X PBS, they were stained using a TRAP assay kit (Cat# 387A-1KT, Sigma-Aldrich, St. Louis, MO, USA) for one hour in the dark at 37 °C. The cells were washed with diH2O, and nuclei were counterstained using hematoxylin solution Gill No 3 (Cat# 387A-1KT, Sigma-Aldrich, St. Louis, MO, USA) for two to three minutes. Cells were washed with alkaline tap water and imaged randomly using the Zeiss Axiovert 10 microscope at 20×/12 Achrostigmat objective. Osteoclasts were identified as cells with three or more nuclei and stained positive for TRAP. Images were processed and counted using ImageJ.

### 4.11. Statistical Analysis

All outliers were removed using Chauvenet’s criterion test. All obtained data were analyzed using the single factor analysis of variance (ANOVA) followed by statistical analyses using the Tukey–Kramer HSD test, student *t*-test, and/or the Fisher’s exact test. Error bars represent standard error of the mean (SEM). Statistical significance was set at *p* < 0.05 and is denoted above error bars as letters, in which “a” represents group one, “b” represents group two, etc. Here, group one is the first bar, group two is the second bar, etc. Bars that display the letter “a” above the error bar denotes that this group is significantly different from group one.

## Data Availability

Any data or material that support the findings of this study can be made available by the corresponding author upon request.
